# The effect of swaddling on infant sleep and arousal: A systematic review and narrative synthesis

**DOI:** 10.3389/fped.2022.1000180

**Published:** 2022-11-30

**Authors:** Allison Dixley, Helen L. Ball

**Affiliations:** Durham Infancy & Sleep Centre, Department of Anthropology, Durham University, Durham, United Kingdom

**Keywords:** infant care [methods], infant sleep, parenting (MeSH), sleep duration, arousal

## Abstract

**Background:**

Western parents swaddle infants to promote sleep and reduce night-waking, however recent evidence of the effects of swaddling on the sleep of healthy infants has not been systematically reviewed.

**Objective:**

To investigate the effect of swaddling on the sleep of infants up to 1 year of age using a narrative synthesis approach.

**Data Sources:**

We systematically searched five academic databases, including the Cochrane trial registry (Pubmed, PsycINFO, Embase, the Cochrane library, and Blackwell Synergy). We manually searched reference lists and citations of included studies and reference lists of existing reviews.

**Study Selection:**

Studies of any type, published since 2007, reporting primary data whose subjects were humans up to 12 months of age with outcome measures relating to the impact of swaddling on sleep and arousal.

**Data Extraction:**

By hand using a customized template. A narrative synthesis is used to present the results.

**Results:**

In total 171 studies were retrieved with 115 studies discarded at title. A further 43 were discarded at abstract, and six were discarded at full text. Two papers were combined as they reported on the same study giving a total of 6 studies that met the inclusion criteria for the review. An evidence hierarchy was used to assess Risk of Bias in the included studies; the results are presented *via* a narrative synthesis. Swaddling was associated with increased duration of quiet sleep in infants and a significantly reduced number of sleep state changes among infants naïve to the intervention.

**Limitations:**

The integrity of the intervention (swaddling) including its baseline characteristics, was defined broadly across the included studies limiting the interpretation and transferability of the results of this review.

**Conclusions:**

Swaddling appears to increase quiet sleep duration in infants and reduces the number of sleep state changes among infants naïve to the intervention. Parents should be made aware that implementing conditions unfavorable to arousability may increase SUDI risk among infant who have not previously been swaddled. This review has relevance for informing future practice recommendations and parent advice as well as in designing future studies.

## Introduction

To explore the effect of swaddling on infant sleep and arousal in infants under 12 months of age we conducted a systematic review of the evidence published over the past 15 years since the review of van Sleuwen et al. (1). This previous review concluded that swaddling infants above the waist increased sleep duration, reduced motor activity and startles, and lowered heart rate, however swaddling was not found to affect the capacity of the infant to respond to stimulation with less-intense auditory stimulation arousing swaddled infants more effectively than non-swaddled infants. The previous review highlighted “the difference in the physiologic effect of swaddling in QS [quiet sleep] and AS [active sleep]” as an avenue requiring more research (1: e1106). Nelson (2) also conducted a systematic review of swaddling to inform nursing practice, however this did not examine effects on infant sleep and arousal. In their meta-analysis, Pease et al. (3) found evidence to suggest risk of SIDS (a subset of SUDI) increases with infant age when infants are swaddled. They identified a twofold risk for infants aged >6 months. However, their review was limited to “SIDS” as an outcome measure, with no measure of sleep and/or arousal processes. The present review sought to fill these knowledge deficits by answering the question, what is the effect of swaddling on infant sleep and arousal? In developing the literature search strategy, we used the PICO framework to ensure comprehensive and bias-free searches.

POPULATION: Infants aged < 1 year.
INTERVENTION: Swaddling.
COMPARISON: Not swaddling.
OUTCOMES: Effects on sleep and arousal.

## Review protocol

Our search aimed to identify studies of any type reporting primary data for human infants <1 year of age, whose outcome measures included information on the impact of swaddling on sleep and arousal. Abstracts were screened by both authors. Studies were excluded if they only examined infants born prematurely (gestational age < 37 weeks), featured a primary intervention other than swaddling, were published before 2007, or were not published in English. To maximise inclusion of relevant studies qualitative and quantitative studies from a range of disciplines were included. This mixed-methods approach is thought to provide a more complete basis for complex clinical and policy decision-making than that currently offered by single method reviews (4). It was particularly appropriate for the present review question, as swaddling is not only a physiological intervention but also a social one; its application differs across and within cultures according to numerous diverse factors such as tradition, health professional advice, policy recommendations, fashion and personality (5–8). The objective of this mixed-methods approach is therefore to integrate a quantitative estimation of swaddling's “effectiveness” *via* measures of infant sleep duration and arousal, with a more qualitative understanding from the perspective of the implementers of the intervention (usually parents), increasing the relevance of the review for policy and practice.

### Search strategy

Our systematic search identified studies published from 2007 to March 2022 covering the same electronic databases as the 2007 review: Pubmed, PsycINFO, Embase, the Cochrane library, and Blackwell Synergy. The search terms used are shown in Table 1. Following standard guidelines for systematic reviews (9) leading journals were hand searched as were the reference lists of those studies meeting the inclusion criteria. Due to lack of resources to undertake translation searches were restricted to English language material. We included all papers that identified any effects of swaddling on infant sleep and arousal even if this topic was not the primary purpose of the paper.

**Table 1:** Search terms used in systematically searching the literature.

Terms and truncations	Terms to be captured in search
infan* OR baby* OR babies OR newborn* OR neonat*	to include infant, infants, baby, baby's, babies, newborn, newborns, neonate, neonatal
AND sleep* OR arous*	sleep, sleeping, arouse, arousal, arousing
AND swaddl* OR restrain* OR wrap*	swaddle, swaddling, swaddled, restrain, restrained, restraining, wrap, wrapping, wrapped

### Data extraction and appraisal

After screening, the first author worked independently to extract data from the included papers. All papers that met the inclusion criteria were read in full, with data extracted by hand using a customized template (Supplementary Appendix 1) (10). Mindful that “the effect of swaddling may be somehow mediated through the modified sleep characteristics in the infants” (11, 12) the data extraction template included factors associated with modified sleep behaviour, such as environmental temperature (9, 13, 14), nocturnal or diurnal setting (15–17) and feeding method (18). Study authors were contacted in cases where information was missing. An appraisal form was used (Supplementary Appendix 2) to assess the quality of each study in terms of internal and external validity.

## Results (narrative synthesis)

In reporting the findings of a systematic mixed-methods review narrative synthesis is an effective approach for explaining potentially conflicting outcomes. Where contradictions arose, our goal was to ascertain epistemological explanations.

### Preliminary synthesis

**Figure 1:**
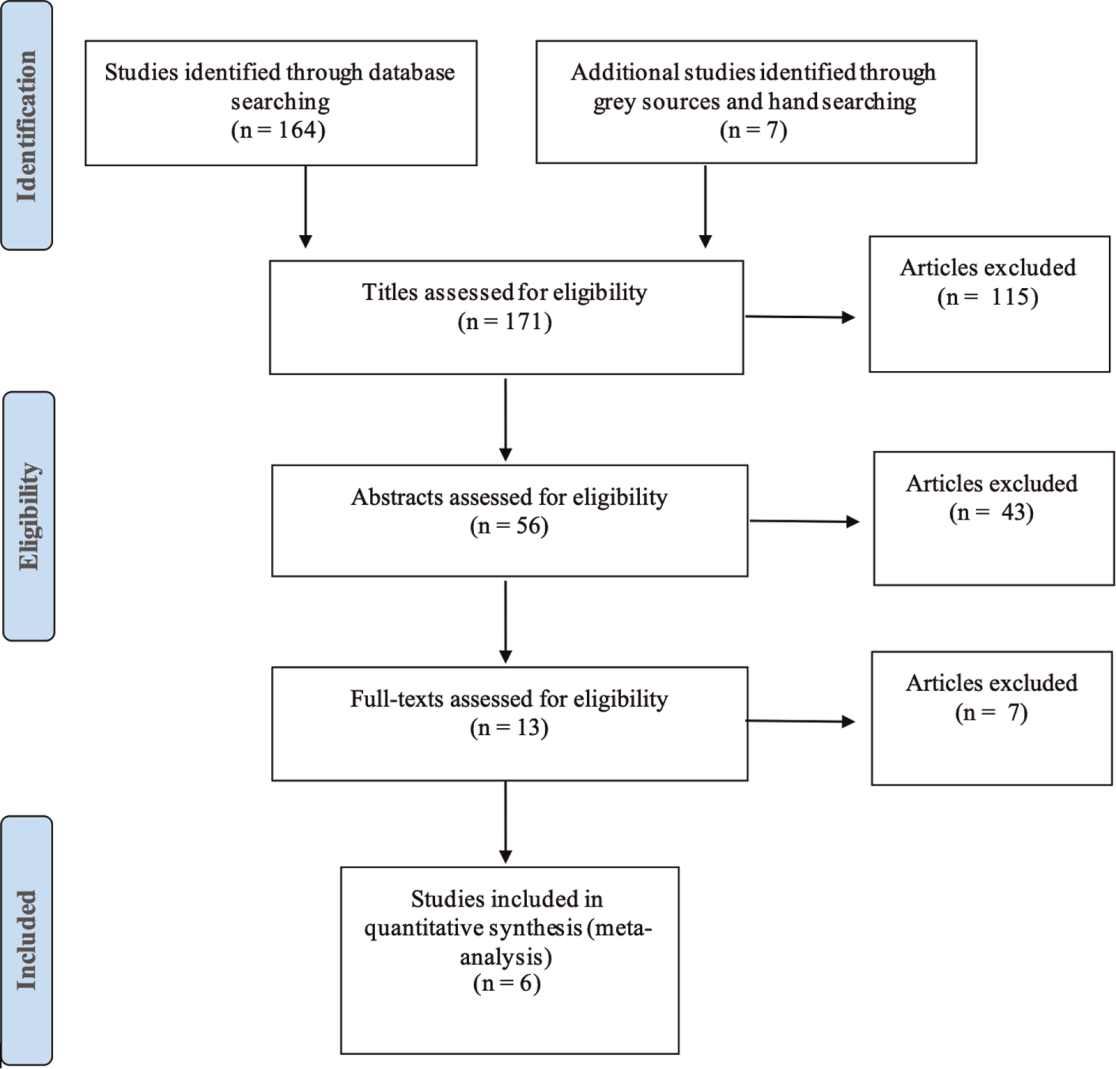
PRISMA flow diagram.

An initial list of 171 studies was retrieved from the initial searches. Studies were assessed against the inclusion criteria with 115 discarded following title review. A further 43 were discarded following a review of abstracts, and 7 were discarded following review of full text. At each stage reviews were conducted by the first author and verified by the second author with discussion of any discrepancies. Where two publications reported on the same study (19, 20) (confirmed by contacting authors) the information extracted was combined as a single study (20), meaning 6 studies met the inclusion criteria (see Figure 1).

The six studies that met the inclusion criteria all used swaddling as their primary intervention and measured infant sleep and arousal outcomes, but varied in their approach to study design and setting. Richardson et al. (20), Meyer and Erler (21), and Narangerel et al. (22) each sought to evaluate the effects of swaddling on different stages of infant arousability, although Narangerel et al. (22) narrowed their focus to respiratory rate and oxygen saturation. The remaining studies, sought to ascertain the effects of swaddling on the temporal nature of infant sleep and arousal. Three studies (20–22) used clinical measures such as polysomnography to assess sleep and arousal while the other three studies (11, 23, 24) were conducted in a home setting and used participant self-report instruments (see Table 2).

**Table 2:** Summary of study designs and key variables.

	Richardson et al 2010	Narangerel et al 2007	Meyer and Erler 2011	McRury and Zolotor 2010	Kelmanson 2013	Öztürk and Bayik 2019
Randomised controlled trial			X	X		
Quasi-experimental	X	X			X	X
Outcomes assessed by	Objective measurement	Objective measurement	Objective measurement	Self report	Self report	Self report
Period of sleep (nocturnal or diurnal) stated	X		X	X	X	X
Study environment	Lab	Home	Lab	Home	Home	Home
Swaddle technique detailed	X	X				
Environmental temperature detailed	X	X				
Feeding method detailed	X					X
Swaddle tightness measured						

The main findings identified from two clinical studies (20, 21) and one self-report study (24) were that swaddling of infants naïve to the intervention was associated with decreased arousal and increased sleep duration. These findings were consistent across nocturnal and diurnal sleep periods. However, two self-report studies did not report any difference in duration of crying or sleeping as a result of swaddling (23) or of any effect of swaddling upon “infant sleep disturbances” (11). The remaining clinical study (22) did not report any measurable effect of swaddling on SaO_2_ or respiratory rates. The authors of two studies (22, 23) cited Type II Errors as explanations for not reporting significant outcomes.

#### Discrepancies between studies

A key factor explaining the discrepancy between study outcomes is the study environment. Two of the three studies detecting measurable outcomes for swaddling (20, 21) were conducted in the controlled environments of sleep laboratories. Those studies that did not report measurable outcomes were conducted in home settings. The only home study to report a measurable outcome (24) did not isolate swaddling but combined it with other interventions simultaneously (holding on side or stomach, shushing, and swinging).

Study population characteristics may also explain the heterogeneity in findings. Narangerel et al. (22) compared swaddled to non-swaddled infants, but did not report whether the swaddled infants were habituated or naïve (unaccustomed) to swaddling. While Richardson et al. (20) found significant outcomes for sleep and arousal in infants naïve to swaddling, this study did not report a significant outcome for routinely swaddled infants. Kelmanson (11) focused only on routinely swaddled infants and did not report significant sleep outcomes. The habituated vs. naïve dichotomy may also explain why McRury and Zolotor (23) did not report significant outcomes, however the latter study did not verify whether the intervention was actually applied. Mothers in the intervention group may have decided against using the intervention, while those who implemented it may have done so using different techniques.

Differences in data collection methods (physiological vs. self-report measures) may also account for the inconsistent outcomes. The self-report studies used bespoke questionnaires (11) and diary methods (23, 24). These are vulnerable to the subjective perceptions of the participants, making it difficult to combine and synthesise the results with those of the other studies. They highlighted a strong discrepancy between maternal perceptions of swaddling effectiveness and physiological markers of effectiveness. Thus, if a sensitivity analysis was conducted in which self-report studies were excluded from the review, swaddling would appear to have an impact upon sleep and arousal in naïve infants. That Narangerel et al. (22), a physiological study whose sample consisted entirely of routinely swaddled infants, did not report significant outcomes would support this hypothesis.

### Robustness of the synthesis

Evidence hierarchy is often used to assess the quality of intervention studies (25). The first author independently applied the hierarchy to determine the appropriateness of study designs for assessing questions of “effectiveness”. In applying this approach to the question of swaddling effectiveness, two studies (11, 23) would be designated “poor quality” in terms of measures of effectiveness, being qualitative studies considered more susceptible to bias than the quasi-experimental studies (20, 22). The randomized trial (21), designated at the top of the hierarchy, would be deemed most robust. However, the objectives of each study are too dissimilar to synthesize in this manner. Narangerel et al. (22) is a particular outlier, focusing specifically on respiratory rate and oxygen saturation, while the other studies examined sleep and arousal more broadly, including maternal perception of swaddling effectiveness.

Varying degrees of confounding were evident across all studies. Only Richardson et al. (20) and Narangerel et al. (22) reduced contamination by accounting for environmental temperature, for instance, which is known to significantly modify an infant's cardiorespiratory and autonomic parameters (26). Moreover, only two studies (20, 24) ascertained infant feeding method. Whether an infant is breast or bottle fed is considered a strong moderating variable with regard to sleep fragmentation (27–29). Potential differences in such important baseline characteristics reduce internal validity (30).

The weakest study in relation to confounding was that of Narangerel et al. (22) which not only failed to allocate participants randomly but also failed to ascertain infant feeding method and whether the sleep studied was nocturnal, diurnal or both. An investigation of sleep and arousal cannot be effective when analyzed in isolation from these key factors (31, 32).

### Selection bias

For most of the included studies it was unclear whether true randomization had been conducted reflecting previous critiques that randomization is inadequately described in most published studies (33–36). Additionally two studies (23, 24) used a recruitment technique that biased the pool of participants towards certain demographics, namely educated, western mothers giving birth in a hospital setting. One study (23) required participants to have “the resources to play a VHS tape/CD at home” and to “contact a research assistant by phone” before being discharged from hospital. Further, mothers who did not speak English or were unable to fill out a diary themselves were excluded. The generalizability of McRury and Zolotor (23) was further weakened by the relatively high attrition rate of 31%. The tendency to drop-out has been associated with ethnicity and low academic achievement (37–39).

### Allocation bias

With regard to allocation bias, those studies which utilized randomization (20, 21, 23, 24) also used comparison groups that were balanced at baseline and thus distributed possible confounding factors evenly. McRury and Zolotor (23) was the strongest study in this respect, being the only one to blind the researchers to control and intervention groups, and to compare demographic and baseline characteristics of the intervention and control participants.

Two studies (11, 22) allocated infants to predefined “natural” intervention and control groups. The outcomes of these studies cannot be confidently attributed to the intervention as their non-randomized nature may have encouraged a tendency to over-estimate effect sizes (40).

However, the greatest threat to the robustness of the synthesis involves the integrity of the intervention. This was problematic across all studies, and it is unclear whether the interventions were similar enough to synthesize: Narangerel et al. (22) for instance, gave a very brief description of the swaddle technique used, namely “two or three layers of cloth that enclosed the trunk and arms up to infant's neck”. Where swaddle technique was described, three (20–22) enclosed the infants' arms within the swaddle. This detail is important as previous research suggests infants swaddled with arms free experience the same startle frequency as unswaddled infants (7). Arm restraint therefore appears to be integral to the effectiveness of the swaddling intervention. The weakest studies, and those most at risk of Type III error, were those lacking any description of swaddle technique (11, 23). While Richardson et al. (20) and Meyer and Erler (21) describe their swaddle techniques in some detail, they along with all other studies, failed to measure or control for swaddle tightness–another important moderating variable discussed below. Richardson et al. (20) merely stated that the infants were “firmly” wrapped.

## Discussion and recommendations

The most contentious factors arising from this review are habituation, sleep stages, sleep environment, swaddle technique and swaddle tightness.

### Habituation (routine vs. non-routine swaddling)

Significant outcomes for swaddling were only detected in non-routinely swaddled infants (20, 21). When samples involved only routinely swaddled infants significant changes in sleep and arousal were not detected (11). The application of routine vs. non-routine sleep interventions has important implications for infant physiology, and therefore health policy. In support of this finding non-routinely swaddled infants have been shown to exhibit higher arousal thresholds (41) and less spontaneous arousability in AS (20), both considered risk factors for SIDS (42). Future studies should further explore habituation to swaddling.

### Sleep states

To date, a largely neglected area of infant sleep research is the interaction between interventions and sleep architecture (changes in sleep states). This review indicates that swaddling significantly reduces the frequency of infant sleep state changes, promoting quiet sleep and sleep efficiency (21).

Previous research has associated a deficiency in acethecholine brain receptors with insufficient arousal (43, 44). It is possible that swaddling, by decreasing the type and number of arousals, may exacerbate this. Furthermore, the arousal threshold from quiet sleep is greater than that from active sleep (21). By promoting greater duration of quiet sleep at a time when arousal mechanisms are underdeveloped, swaddling may impair an infant's ability to handle a regulatory crisis, such as prolonged apnea. Future research should therefore examine the effect of swaddling on the sequence and duration of infant sleep states.

### Sleep environment

Both studies conducted in sleep laboratories (20, 21) found swaddling to have measurable outcomes, and there is evidence from other sleep studies that the study environment is an important moderating variable (45). For instance, swaddled infants have been shown to demonstrate increased responsiveness to environmental auditory stress compared to the unswaddled condition (46). Another facet of environment, bedsharing, has also been shown to influence infant sleep and arousal (28, 29, 47). Bedsharing infants experience significantly less time in deep sleep and significantly more shifts in sleep stages than solitary sleeping infants (48). Despite these factors, none of the studies in the present review detailed whether bedsharing or co-sleeping (parent sharing room with infant) was a factor considered in their analysis.

### Swaddle tightness

Motor restraint is a key component of swaddling, fundamental to the intervention. It has been suggested that the sedative effects of swaddling are “triggered by very specific stimuli delivered above a certain threshold” (37, 49). This threshold may, in part, be determined by tightness. Indeed, the decreased arousability procured through swaddling is unlikely to be explained merely by the material's constant stimulation of the skin and thermal receptors, as one study in the review (20) controlled for these factors and still found decreased arousability in swaddled infants. Yet despite the potential importance of swaddle tightness, none of the studies attempted to measure or control it, weakening the value of the studies reviewed. Swaddle tightness is an elusive variable, not only across swaddling studies, but also within health policy. Australian guidelines (12) advise that swaddles “should be firm, to prevent loose wrapping becoming loose bedding. However, the wrap should not be too tight and must allow for hip and chest wall movement.” “New guidance from the American Academy of Pediatrics does not mention tightness at all (50). Earlier guidance provided slightly more detail: The swaddle should not be too tight or make it hard for your baby to breathe or move their hips” (51).

That swaddled infants demonstrate heightened responsiveness to environmental auditory stress (26, 46) supports the hypothesis that tightness may be an important factor in the effectiveness of swaddling. It is possible, for instance, that the tightness of the swaddle numbs tactile sensory receptors prompting a compensatory increase in auditory sensory reception.

There is also evidence to suggest that swaddle tightness may impact cardiorespiratory function. This has implications for SIDS, as abnormalities in cardiorespiratory function have been shown to impact the arousal response needed to regulate breathing (41, 52, 53). Unless very loose, swaddling increases thoracic compression (54) which leads to a reduction in oxygen saturation and a consequent increase in respiratory rate and cardiac activity (21). These physiological changes may explain why swaddling has been associated with an increased risk of acute respiratory infection (1, 55). The finding that swaddling has little or no effect on respiratory rate applies only to routinely swaddled infants (22) contrasting with earlier studies (56) and with the previous review (1). The implications of this are important as impaired respiratory and heart rates are associated with arousal difficulties, a key mechanism proposed in the triggering of SIDS (41). An investigation which explicitly examines swaddle tightness would therefore be of benefit, particularly with regard to the formation of policy guidelines for cardio-respiratory compromised infants.

### Limitation of review

Only six studies met the inclusion criteria, and no consistent outcomes were revealed. As the integrity of the intervention varied across all studies reviewed, including baseline characteristics, interpretation and transferability of the results should be treated with caution.

## Conclusions

Swaddling was a complex intervention to systematically review due to the variable nature of its application, and the considerable heterogeneity across studies regarding participant characteristics (e.g., feeding method), environment (e.g., laboratory or home) and outcomes measured (e.g., sleep, arousal, or facets of both). By combining physiological and self-report studies, this review provides an understanding of swaddling under differing research conditions.

This review supports the possibility that, by producing conditions unfavorable to arousability, swaddling may increase SIDS risk in infants that are naïve to the experience (3, 20). This argument is strengthened by the finding that swaddling significantly reduces the frequency of sleep state transitions and promotes quiet sleep. Given that swaddling is typically used during the critical period in which infants are most vulnerable to SIDS, namely <6 months of age (52, 57) further research into the effect of swaddling on infant sleep and arousal, particularly for swaddle-naïve infants, is strongly recommended.

## Data Availability

The original contributions presented in the study are included in the article/Supplementary Material, further inquiries can be directed to the corresponding author/s.
