# Supplementary material for: The effect of swaddling on infant sleep and arousal: A systematic review and narrative synthesis
Source: Front Pediatr. 2022 Nov 30;10:1000180. doi: 10.3389/fped.2022.1000180 (PMC9748185; doi:10.3389/fped.2022.1000180)
Supplement: Supplementary file 1 [file Datasheet1.docx]

## Appendix 1. Systematic review, study extraction template

| *Data to be extracted* | *Notes* |
| --- | --- |
| **BASIC DESCRIPTIVE INFORMATION** |  |
| Title of study |  |
| Author |  |
| Year of publication |  |
| Study objective as stated by authors |  |
| Research setting |  |
| Data analysis (time/event sampling) |  |
| Discipline |  |
| Synopsis |  |
| **SPIDER** |  |
| Sample |  |
| Phenomenon of Interest (what was measured?) |  |
| Design (how was it measured?) |  |
| Evaluation (outcomes) |  |
| Research type (qual/quant/mixed) |  |
| **DETAILED METHODOLOGICAL INFORMATION** |  |
| Theoretical position (inductive/deductive/narrative-evolving) |  |
| Type of outcome observed  (i.e. continuous such as sleep, or binary "events" such as vocalisations, awakenings). |  |
| Mode of data management |  |
| Editing/cleaning of data |  |
| Use of pre-established coding scheme or codes derived from the data? |  |
| Coding software used |  |
| Is coding microanalytic or macroanalytic |  |
| Analytic plan |  |
| Explanation of findings |  |
| Ethical considerations |  |
