# Supplementary material for: The effect of swaddling on infant sleep and arousal: A systematic review and narrative synthesis
Source: Front Pediatr. 2022 Nov 30;10:1000180. doi: 10.3389/fped.2022.1000180 (PMC9748185; doi:10.3389/fped.2022.1000180)
Supplement: Supplementary file 2 [file Datasheet2.docx]

## Study appraisal template

| Study title |  |
| --- | --- |
| Author |  |
| SAMPLE |  |
| Was method of participant selection vulnerable to selection bias? |  |
| PHENOMENON OF INTEREST |  |
| Allocation bias |  |
| Risk of confounding |  |
| DESIGN |  |
| Does the study have a clearly stated aim/s? |  |
| Is data collection methodology appropriate for the purpose and aims of the study? |  |
| Is there a clear data collection protocol? |  |
| Is there a clearly identified comparison group, identical in all aspects other than exposure? |  |
| Is there a clear coding protocol? |  |
| Risk of researcher bias |  |
| Use of inter-rater reliability |  |
| Funding |  |
| Conflicts of interest |  |
| EVALUATION (OUTCOMES) |  |
| Is there a clear statement of findings? |  |
| Are the basic data adequately described? |  |
| Where the aims of the study met? |  |
| Power issues |  |
| Missing data |  |
| Did they identify new areas where research is necessary |  |
| Contribution to knowledgebase |  |
| RESEARCH TYPE (QUAL/QUANT/MIXED) |  |
| Does the study have a stated methodology? |  |
| If so, is it adhered to? |  |
